# Examining prevalence and correlates of smoking opioids in British Columbia: opioids are more often smoked than injected

**DOI:** 10.1186/s13011-021-00414-6

**Published:** 2021-10-18

**Authors:** Stephanie Parent, Kristi Papamihali, Brittany Graham, Jane A. Buxton

**Affiliations:** 1 Faculty of Medicine, Queen’s University, Kingston, Ontario Canada; 2 Harm Reduction Services, BC Centre for Disease Control, 655 West 12th Avenue, Vancouver, BC V5Z 4R4 Canada; 3 School of Population and Public Health, University of British Columbia, 655 West 12th Avenue, Vancouver, BC V5Z 4R4 Canada

**Keywords:** Opioids, Mode of administration, Smoking, Inhaling, People who use drugs

## Abstract

**Background:**

British Columbia (BC) is in the midst of an opioid overdose crisis. Since 2017, smoking illicit drugs has been the leading mode of drug administration causing overdose death. Yet, little is known about people who smoke opioids, and factors underlying choice of mode of administration. The study objectives are to identify the prevalence and correlates associated with smoking opioids.

**Methods:**

The Harm Reduction Client Survey is a monitoring tool used by the BC Centre for Disease Control since 2012. This survey is disseminated to harm reduction sites across BC to understand drug use trends and drug-related harms. We examined data from the survey administered October–December 2019 and performed descriptive, univariate, and multivariate analyses to better understand factors associated with smoking opioids.

**Results:**

A total of 369 people who used opioids in the past 3 days were included, of whom 251 (68.0%) reported smoking opioids. A total of 109 (29.5%) respondents experienced an overdose in the past 6 months; of these 79 (72.5%) smoked opioids. Factors significantly associated with smoking opioids were: living in a small community (AOR =2.41, CI =1.27–4.58), being a woman (AOR = 1.84, CI = 1.03–3.30), age under 30 (AOR = 5.41, CI = 2.19–13.40) or 30–39 (AOR = 2.77, CI = 1.33–5.78) compared to age ≥ 50, using drugs alone (AOR = 2.98, CI = 1.30–6.83), and owning a take-home naloxone kit (AOR = 2.01, CI = 1.08–3.72). Reported use of methamphetamines within the past 3 days was strongly associated with smoking opioids (AOR = 6.48, CI = 3.51–11.96).

**Conclusions:**

Our findings highlight important correlates associated with smoking opioids, particularly the recent use of methamphetamines. These findings identify actions to better respond to the overdose crisis, such as targeted harm reduction approaches, educating on safer smoking, advocating for consumption sites where people can smoke drugs, and providing a regulated supply of opioids that can be smoked.

## Background

In recent years, opioid-related overdoses have significantly increased and devastated communities across North America [1]. Fentanyl, a synthetic opioid recently introduced in the illicit drug supply, is one of the main factors driving this alarming increase in overdoses and death [2]. British Columbia (BC) is one of the hardest hit regions with drug-related deaths increasing from 211 in 2010 to 1726 in 2020 [3, 4]. This increase in deaths led to the declaration of a public health emergency by the Provincial Health Officer in 2016, and this status remains active as of the writing of this article [5].

Various interventions aimed at reducing overdose deaths have been developed. For example, in 2012, the BC Centre for Disease Control (BCCDC) introduced a take-home naloxone program; anyone at risk of experiencing or witnessing an overdose can access free naloxone kits and training on how to use it. To date, more than one million naloxone kits have been shipped to community sites for distribution [6]. Another initiative aimed at preventing overdose-related deaths in Canada is the Good Samaritan Drug Overdose Act [7]. This Act, enacted in May 2017, provides legal protection for simple drug possession for people at the scene of an overdose, with the goal of encouraging professional help-seeking during an overdose. However, awareness and effectiveness of the Act among a population historically mistrustful of authority is equivocal [8]. It is also worth noting that BC was the first jurisdiction in North America to implement a supervised injection site in 2003, and the success of this intervention in preventing overdoses is well established, though barriers to access remain [9]. In light of the overdose emergency, in 2016, the BC Ministry of Health mandated that all health authorities provide overdose prevention services, and as of May 2021, there are over 40 overdose prevention services and supervised consumption sites in BC [10, 11].

Yet, despite these and other initiatives, overdoses and related deaths in BC continue to increase: 2020 marked the year with the highest illicit drug toxicity ever recorded [4]. While the COVID-19 pandemic certainly played a role in this increase [12], more inquiry into the factors causing these deaths and how they can be prevented is necessary.

Mode of drug administration is one often overlooked factor that may be contributing to overdoses. Until 2017, injection drug use was the leading mode of consumption among overdose deaths in BC [13]. However, this changed in 2017, when smoking illicit drugs became the leading mode of drug administration among overdose deaths [13]. Smoking continued to be the leading mode of drug administration causing death in the following years, while injecting declined from 37% in 2016 to 25% in 2019 [13]. Qualitative data from populations who smoke crack indicate that participants perceived that smoking drugs was safer than injecting [14]. In addition, people who preferred non-injection modes of drug administration were less likely to carry a naloxone kit [15]. Yet, little is known about people who smoke opioids, and the factors underlying choice of drug mode of administration. Uncovering these factors may help in refining harm reduction strategies to improve the response to the overdose crisis. Thus, the objectives of this study are to identify the prevalence and correlates associated with smoking opioids.

## Methods

### Study design

The Harm Reduction Client Survey (HRCS) is a monitoring tool used by the BCCDC since 2012 and has been described in detail elsewhere [15–19]. To summarize briefly, we worked in collaboration with regional harm reduction service coordinators to identify harm reduction supply distribution sites across all regional health authorities in BC. Participants were included in the survey if they were aged > 18; self-reported using illicit drugs in the past 6 months; and provided verbal consent for participation [18]. Participants were recruited from 22 sites by trained staff and volunteers based on willingness to participate, and the survey was administered in-person at the harm reduction site by the site staff and volunteers. All participants consented to being included in the study, and were paid $10 CAD, and sites were also paid $5 CAD per participant. Completed paper surveys were mailed to the BCCDC.

The aim of this cross-sectional survey was to understand drug use patterns in order to optimize harm reduction strategies and services. For this analysis, we used an existing survey and included all participants who reported using any opioids in the past 3 days, including hydromorphone, oxycodone, morphine, heroin, or fentanyl, but excluding methadone and Suboxone. We used data from the 2019 HRCS which was administered between October and November 2019.

### Study variables

The main outcome variable was “smoking opioids” (yes/no), a composite variable created by amalgamating participants who had answered “yes” to smoking hydromorphone, oxycodone, morphine, fentanyl, and/or heroin in the past 3 days. The main explanatory variables included demographic and substance use variables. Demographic variables were urbanicity of community (small urban/rural communities, medium/large urban cities), defined according to the BC Ministry of Health’s classification system that combines indicators set by Statistics Canada such as population density and proximity to urban areas [20–22]; Health Authority (Fraser Health, Interior Health, Island Health, Northern Health, Vancouver Coastal Health); gender identity (man, woman, transgender and gender expansive, unknown); age (under 30, 30–39, 40–49, 50 and over, unknown); regular housing (yes, no, unknown), defined as having answered “yes” to living in a private residence or other residence such as hotel, motel, or rooming house; employment, defined as having answered “yes” to working either part-time, full-time, or as a paid volunteer (yes, no, unknown). Substance use variables in the past 6 months were: experiencing an overdose (yes, no, unknown); witnessing an opioid overdose (yes, no, unknown); use of observed consumption services (OCS) [11], which includes overdose prevention services and supervised consumption sites (yes, no, unknown); owning a take-home naloxone kit (yes, no, unknown); using drugs alone (yes – includes those who answered occasionally, often, or always-, no, unknown). Substance use in the past 3 days included: cocaine; crack; methamphetamines; and opioid agonist treatment (OAT) (yes, no, unknown). Participants were classified as having used OAT in the past 3 days if they reported use of methadone and/or buprenorphine in the past 3 days. Individuals who had missing responses or who answered, “prefer not to say” to the dependent variables were included in the analyses and categorized as “unknown”.

### Data analyses

First, we calculated descriptive statistics for all variables of interests, stratified by “smoking opioids”. To estimate the unadjusted and adjusted effects of factors on smoking opioids, bivariable analyses were used to explore associations between conceptually relevant explanatory variables and smoking opioids. Variables with a *p*-value < 0.25 in bivariable analyses were included in the multivariable analysis. A multivariable logistic regression model was constructed to derive adjusted effects of explanatory variables on smoking opioids, with those who did not smoke opioids as the reference group. Adjusted odds ratio (AOR), 95% confidence intervals (CI), and *p*-values are reported, with *p*-values < 0.05 considered statistically significant. All statistical analyses were conducted using R Version 4.0.2. This study was approved by the University of British Columbia Office of Behavioural Research Ethics (#H07–00570).

## Results

A total of 621 participants responded to the 2019 HRCS. Of these, 369 participants indicated they had used opioids in the past 3 days and were included in this study. Heroin (73.7%) and fentanyl (76.7%) were the opioids with the most reported use. Of the 5 BC regional health authorities, the largest proportion of participants (33.9%) was from Fraser Health (Fraser Health serves 1.8 million people, 35% of the total BC population) [23], and only 10.8% were from Island Health (Island Health serves 850,000 people, 17% of the total BC population) [24]. The majority of participants identified as man (59.9%), while 37.1% identified as woman and 1.9% identified as other genders, which included trans man, trans woman, and gender non-conforming participants. The largest proportion of participants were aged 30–39 (34.4%). Table 1 outlines summary characteristics of the study sample.

**Table 1:** Summary characteristics of the study population (row percent)

	Did not smoke opioids (*N* = 118)	Smoked opioids (*N* = 251)	Total (*N* = 369) (column %)	*p* value
**Health Authority**				0.142
Fraser	47 (37.6%)	78 (62.4%)	125 (33.9%)	
Interior	16 (26.7%)	44 (73.3%)	60 (16.3%)	
Island	9 (22.5%)	31 (77.5%)	40 (10.8%)	
Northern	15 (24.6%)	46 (75.4%)	61 (16.5%)	
Vancouver Coastal	31 (37.3%)	52 (62.7%)	83 (22.5%)	
**Urban/Rural**				
Medium/Large Urban	96 (36.4%)	168 (63.6%)	264 (71.5%)	
Small Urban or rural	22 (21.0%)	83 (79.0%)	105 (28.5%)	
**Gender identity**				0.129
Woman	34 (24.8%)	103 (75.2%)	137 (37.1%)	
Man	80 (36.2%)	141 (63.8%)	221 (59.9%)	
Transgender and gender expansive	2 (28.6%)	5 (71.4%)	7 (1.9%)	
Unknown	2 (50.0%)	2 (50.0%)	4 (1.1%)	
**Age**				< 0.001
29 and under	15 (18.8%)	65 (81.2%)	80 (21.7%)	
30–39	34 (26.8%)	93 (73.2%)	127 (34.4%)	
40–49	33 (37.5%)	55 (62.5%)	88 (23.8%)	
50 and over	32 (48.5%)	34 (51.5%)	66 (17.9%)	
Unknown	4 (50.0%)	4 (50.0%)	8 (2.2%)	
**Regular Housing**				0.691
Yes	76 (33.5%)	151 (66.5%)	227 (61.5%)	
No	39 (30.0%)	91 (70.0%)	130 (35.2%)	
Unknown	3 (25.0%)	9 (75.0%)	12 (3.3%)	
**Employed**				0.044
No	88 (30.9%)	197 (69.1%)	285 (77.2%)	
Yes	29 (40.8%)	42 (59.2%)	71 (19.2%)	
Unknown	1 (7.7%)	12 (92.3%)	13 (3.5%)	
**Experienced an overdose**^**a**^				0.414
No	83 (33.5%)	165 (66.5%)	248 (67.2%)	
Yes	30 (27.5%)	79 (72.5%)	109 (29.5%)	
Unknown	5 (41.7%)	7 (58.3%)	12 (3.3%)	
**Witnessed an opioid overdose**^**a**^				0.011
No	39 (39.8%)	59 (60.2%)	98 (26.6%)	
Yes	59 (26.2%)	166 (73.8%)	225 (61.0%)	
Unknown	20 (43.5%)	26 (56.5%)	46 (12.5%)	
**Used drugs alone**				0.007
No	19 (45.2%)	23 (54.8%)	42 (11.4%)	
Yes	91 (29.0%)	223 (71.0%)	314 (85.1%)	
Unknown	8 (61.5%)	5 (38.5%)	13 (3.5%)	
**Used observed consumption services**^**a**^				0.503
No	55 (31.1%)	122 (68.9%)	177 (48.0%)	
Yes	52 (31.3%)	114 (68.7%)	166 (45.0%)	
Unknown	11 (42.3%)	15 (57.7%)	26 (7.0%)	
**Opioid agonist therapy (OAT)**^**b**^				0.188
No	87 (34.1%)	168 (65.9%)	255 (69.1%)	
Yes	31 (27.2%)	83 (72.8%)	114 (30.9%)	
**Own take-home naloxone**				0.017
No	37 (43.5%)	48 (56.5%)	85 (23.0%)	
Yes	74 (27.7%)	193 (72.3%)	267 (72.4%)	
Unknown	7 (41.2%)	10 (58.8%)	17 (4.6%)	
**Hydromorphone**^**b**^				0.444
No	114 (32.4%)	238 (67.6%)	352 (95.4%)	
Yes	4 (23.5%)	13 (76.5%)	17 (4.6%)	
**Oxycodone**^**b**^				0.943
No	116 (32.0%)	247 (68.0%)	363 (98.4%)	
Yes	2 (33.3%)	4 (66.7%)	6 (1.6%)	
**Morphine**^**b**^				0.006
No	86 (28.8%)	213 (71.2%)	299 (81.0%)	
Yes	32 (45.7%)	38 (54.3%)	70 (19.0%)	
**Heroin**^**b**^				< 0.001
No	48 (49.5%)	49 (50.5%)	97 (26.3%)	
Yes	70 (25.7%)	202 (74.3%)	272 (73.7%)	
**Fentanyl**^**b**^				0.012
No	37 (43.0%)	49 (57.0%)	86 (23.3%)	
Yes	81 (28.6%)	202 (71.4%)	283 (76.7%)	
**Methamphetamines** ^**b**^				< 0.001
No	52 (63.4%)	30 (36.6%)	82 (22.2%)	
Yes	66 (23.0%)	221 (77.0%)	287 (77.8%)	
**Crack**^**b**^				0.629
No	97 (32.6%)	201 (67.4%)	298 (80.8%)	
Yes	21 (29.6%)	50 (70.4%)	71 (19.2%)	
**Cocaine**^**b**^				0.655
No	90 (32.6%)	186 (67.4%)	276 (74.8%)	
Yes	28 (30.1%)	65 (69.9%)	93 (25.2%)	

^a^Last 6 months

^b^Past three days

**Fig. 1:**
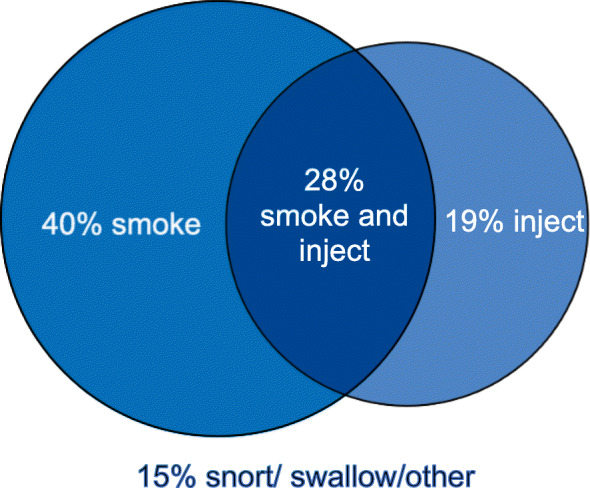
Mode of opioid administration. Legend: 40% of participants exclusively smoke opioids, 19% exclusively inject, 28% both smoke and inject, while 15% use other mode of drug administration

The majority of participants (68.0%, *n* = 251) reported smoking opioids in the past 3 days, and 147 (39.8% of total participants) exclusively smoked opioids, while 68 (18.4% of total participants) exclusively injected (Fig. 1). Participants may use more than one mode of administration, and in our study, 104 (28.2% of total participants) of the total reported both smoking and injecting. A minority of participants (14.9% of total participants, *n* = 55) reported snorting, swallowing, or “other” as mode of drug administration (Fig. 1). Across the study population, the opioids reported used were mostly fentanyl and heroin, with a very small proportion of participants consuming hydromorphone or oxycodone (Table 1). The majority of participants (77.8%) also indicated consuming methamphetamines in the past 3 days.

A total of 109 (29.5%) participants experienced an overdose in the past 6 months; of these, 79 (72.5%) report smoking opioids in past 3 days. A majority of respondents who had used opioids in the past 3 days (85.1%) reported using drugs alone, had not used OAT in the past 3 days (69.1%), and owned take-home naloxone (72.5%).

Table 2 presents the results from the adjusted odds ratio for the main outcome variable (smoking opioids in the past 3 days). The variables regular housing, experienced an overdose in the past 6 months, frequenting OCS, using crack, and using cocaine were excluded from the multivariable analysis due to statistical non-significance. Participants who used methamphetamines had 6 times higher odds of smoking opioids (AOR = 6.48, CI = 3.51–11.96). Other variables associated with smoking opioids were living in a small urban or rural area (AOR = 2.41, CI = 1.27–4.58), identifying as a woman (AOR = 1.84, CI = 1.03–3.30), age under 30 (AOR = 5.41, CI = 2.19–13.40) or 30–39 (AOR = 2.77, CI = 1.33–5.78) compared to age ≥ 50, using drugs alone (AOR = 2.98, CI = 1.30–6.83), and owning a take-home naloxone kit (AOR = 2.01, CI = 1.08–3.72).

**Table 2:** Adjusted odds ratio and 95% confidence intervals for correlates of smoking opioids

Variable	Adjusted Odds Ratio (AOR)	95% Confidence Interval (CI)	***p -*** value
**Urban/Rural**			
Medium/Large Urban	1.00	Reference	Reference
Small Urban/Rural	2.41	1.27–4.58	0.01*
**Gender identity**			
Man	1.00	Reference	Reference
Woman	1.84	1.03–3.30	0.04*
Transgender and Gender expansive	1.29	0.21–8.01	0.78
Unknown	0.33	0.02–5.43	0.44
**Age**			
Under 30	5.41	2.19–13.40	< 0.01*
30–39	2.77	1.33–5.78	0.01*
40–49	1.51	0.71–3.21	0.29
50 and over	1.00	Reference	Reference
Unknown	0.53	0.08–3.39	0.5
**Employed**			
Yes	1.00	Reference	Reference
No	1.05	0.55–2.02	0.89
Unknown	16.49	1.18–230.45	0.04
**Witnessed an opioid overdose**			
No	1.00	Reference	Reference
Yes	1.42	0.77–2.60	0.26
Unknown	0.86	0.33–2.29	0.77
**Using drugs alone**			
No	1.00	Reference	Reference
Yes	2.98	1.30–6.83	0.01*
Unknown	0.57	0.12–2.68	0.48
**Used OAT**			
No	1.00	Reference	Reference
Yes	1.75	0.97–3.15	0.06
**Own take-home naloxone**			
No	1.00	Reference	Reference
Yes	2.01	1.08–3.72	0.03*
Unknown	1.30	0.32–5.26	0.71
**Methamphetamines**			
No	1.00	Reference	Reference
Yes	6.48	3.51–11.96	< 0.01*

## Discussion

Our findings indicate that smoking was the preferred main mode of opioid administration in this sample of participants who frequented harm reduction sites in BC. People who smoked opioids were younger (39 years old or less) than people who did not smoke, with those under 30 especially more likely to smoke opioids. This may be because those initiating opioid use primarily follow the trajectory from non-injection methods to injection [25, 26]; thus, younger age groups in the current study may not have yet transitioned to injecting. Data from the BC Coroners Service indicate that young people who use drugs are vulnerable to overdose death. In BC in 2020, 42% of overdoses causing death were in people aged less than 39, including 324 deaths in people aged less than 30 [4]. The median age at time of overdose death was 43, and 70,000 potential years of life have been lost to overdose in BC in 2020 [27]. The most common modes of consumption resulting in overdose death in the under 30 age group was smoking [3]. These statistics, coupled with our findings, point to the dire need to focus harm reduction approaches to target young people who smoke opioids.

In our study, nearly two thirds of participants living in medium or large urban centres smoked opioids, while 79% of those living in small urban or rural centres smoked opioids. Those living in a small urban/rural centre had almost 2.5 times the odds of smoking opioids when compared to those living in larger centres. This could be due to the lower availability and acceptability of needle distribution programs in these areas, where people who use drugs are more likely to be recognized and ostracized [28, 29]. In addition, stigma against people who use drugs is pervasive in rural settings [29]. This holds particularly true for people who inject drugs, as injection drug use carried greater stigma in rural settings, compared to smoking or ingesting drugs [29]. In this context, it is not surprising that people who use drugs in rural setting have higher odds of smoking opioids.

People who used drugs alone were three times more likely to smoke opioids. In comparison, 29% of people who did not smoke opioids used alone. This is a concerning finding, as using alone eliminates the opportunity for someone to respond in case of an overdose. For instance, the majority of fatal overdoses in BC occurred inside private residences by people who used drugs alone, and this held true across health authorities and age groups [3, 4]. A factor explaining the relationship between using alone and smoking may be that many OCS in BC do not currently allow for smoking [30]. OCS allow for timely overdose response and prevent death [4, 9]. In order to support the high proportion of people who smoke alone, it is crucial to consider expanding the availability of inhalation rooms in OCS, and spread awareness of apps like Lifeguard [31] and BeSafe [32], that allow people to have an emergency safety plan or alert emergency services in the event of an overdose while using alone. In addition, it is important to address housing policies which create barriers to access for people who are willing to witness a resident in a hotel or supportive housing using substances and respond to an overdose should it occur. The expansion of these strategies could contribute to reducing overdose deaths among those who prefer to smoke opioids.

Participants who smoked opioids were twice as likely to own a naloxone kit. In contrast, in a BC study on correlates of naloxone kit possession, people who preferred smoking/snorting or inhaling any drugs were less likely to own a kit than people who injected [15]. The higher likelihood of owning a naloxone kit among participants in our study may be specific to people who use opioids, who are aware of the toxic illicit drug supply containing fentanyl and its analogues.

Participants who had used both methamphetamines and opioids in the past 3 days had 6 times the odds of smoking opioids when compared to those who did not use methamphetamines. While the prevalence of opioid use has remained high in BC since 2012, the use of crystal meth has steadily increased since 2012, surpassing non-fentanyl opioids in 2017 [4, 20]. Opioids and crystal meth are often used at the same time or immediately before or after one another, increasing toxicity and risk of overdose [33, 34]. Studies are starting to explore the motivations for using both drugs, such as managing symptoms of withdrawal and desire for an enhanced high [34]. Future studies can further explore the motivations and pattern of co-use of opioids and crystal meth, in order to target harm reduction and education programs to prevent harms that may arise from this pattern of use.

The motivation behind smoking as choice of drug administration is unclear, but the literature offers hypothesis such as fear of needles, fear of bloodborne illnesses, and perceived risk of injecting heroin as damaging to the immune system, as important motivators to choose smoking over injecting [35]. In addition, there were perceptions among people who smoke drugs that non-injecting modes carry less risk of overdose than injecting [14, 35, 36]. This misconception is concerning, particularly with the rise in fentanyl and its high potency and rapid-acting onset of effects [37, 38]. In addition, a retrospective cohort study of calls to the US Poison Centre Program reports that inhaling and injecting opioids carried a similar risk of mortality or life-threatening symptoms when compared to ingesting (RR = 2.24 and 2.60 respectively) [39]. Another study comparing blood oxygen levels (oxygen saturation) in people who injected (*N* = 12) and inhaled (*N* = 10) heroin found that oxygen saturation dropped significantly within 15 min, unrelated to heroin dose, with no difference between people who injected or inhaled [40]. In addition, severity of dependence in people who are new to using is greater in heroin smokers than non-smokers [36], again challenging the misconception that smoking opioids is less harmful than injecting. Thus, the literature is unequivocal that smoking opioids does not provide any additional safety over injecting with regards to morbidity and mortality associated with opioid use [41]. Increasing education on overdose risks when smoking as well as injecting opioids may help increase awareness of risk among people who use drugs.

### Strength and limitations

The current study strengths include providing important insight into an overlooked aspect of the overdose crisis, that is, that smoking opioids is the dominant pattern of use among participants who used opioids, and identifying factors associated with smoking opioids. In addition, this study provides an overview of patterns across BC, a necessary point of view considering the majority of studies with people who use drugs focus on large urban centres such as Vancouver. Limitations of this cross-sectional study include the inability to infer causal and temporal relationships, and the possibility of recall bias. We minimized recall bias by asking participants about their drug use patterns in the past 3 days. Since we are asking about drug use, social desirability bias is a concern, but validation studies report that survey responses of people who use drugs are accurate [42, 43]. Lastly, the sample of this study were participants who used harm reduction sites, and who agreed to participate in the study. These participants may be different from people use opioids but do not use harm reduction services, and from those who did not agree to participate. As such, there is the risk of selection bias, and the results of the study must be interpreted with this in mind.

## Conclusion

Our findings highlight important correlates associated with smoking opioids, particularly the concurrent use of methamphetamines. These findings can support concrete actions to better respond to the overdose crisis, such as targeting harm reduction approaches, educating on the risks of smoking opioids, advocating for consumption sites where people can smoke drugs, as well as providing a safer opioid supply with known content that can be smoked. It is worth noting that as of the writing of this article, the BC Ministry of Mental Health and Addictions announced funding for 12 new inhalation services in communities “hit hardest by the overdose crisis” [44]. Future studies could use qualitative methods to better understand the motivations behind smoking as the preferred mode of drug administration. Understanding how the patterns of drug administration influence mortality is another potential area of future research and an important step to address the overdose crisis currently occurring across North America.

## Data Availability

All data generated or analysed during this study are included in this published article.

## Abbreviations

AOR: Adjusted odds ratio
BC: British Columbia
BCCDC: British Columbia centre for disease control
CI: Confidence intervals
HRCS: Harm reduction client survey
OAT: Opioid agonist treatment
OCS: Observed consumption services
THN: Take-home naloxone
